# *Clostridioides difficile* infection: microbe-microbe interactions and live biotherapeutics

**DOI:** 10.3389/fmicb.2023.1182612

**Published:** 2023-05-09

**Authors:** Ruojun Wang

**Affiliations:** Department of Molecular Biology, Princeton University, Princeton, NJ, United States

**Keywords:** *Clostridioides difficile*, *Clostridioides difficile* infections (CDI), gut microbiota, microbe-microbe interactions, live biotherapeutic products (LBP), fecal microbiota transplantation (FMT)

## Abstract

*Clostridioides difficile* is a gram-positive, spore-forming, obligate anaerobe that infects the colon. *C. difficile* is estimated to cause nearly half a million cases in the United States annually, with about 29,000 associated deaths. Unfortunately, the current antibiotic treatment is not ideal. While antibiotics can treat the infections, they also disrupt the gut microbiota that mediates colonization resistance against enteric pathogens, including *C. difficile*; disrupted gut microbiota provides a window of opportunity for recurrent infections. Therefore, therapeutics that restore the gut microbiota and suppress *C. difficile* are being evaluated for safety and efficacy. This review will start with mechanisms by which gut bacteria affect *C. difficile* pathogenesis, followed by a discussion on biotherapeutics for recurrent *C. difficile* infections.

## Introduction

*Clostridioides difficile* is a spore-forming bacterium that infects the large intestine. Contact with *C. difficile* results in various outcomes: from no colonization to asymptomatic carriages, from mild diarrhea to life-threatening complications (Rupnik et al., 2009; Crobach et al., 2018). *Clostridioides difficile* infection (CDI) is one of the most common causes of healthcare-associated infections in the United States: the estimated burden of CDI was 462,100 cases in 2017, of which roughly half were healthcare-associated cases (Guh et al., 2020). The cumulative incidence of CDI ranges from 1.12 to 631.80 cases per 100,000 population per year based on a meta-analysis of the global CDI burden (Balsells et al., 2019).

One of the most challenging tasks in treating CDI is managing recurrent infections. Vancomycin and metronidazole have been used as first-line CDI treatments for decades. However, they also disrupt the commensal gut microbes; CDI recurrence occurs in at least 20–30% of cases within 60 days of either treatment, potentially involving failure to promptly restore the gut microbial community that defends against enteric pathogens (McFarland et al., 2002; Pépin et al., 2006; DuPont, 2011; Louie et al., 2011). In 2011, fidaxomicin, a narrow-spectrum antibiotic, was approved for the treatment of CDI. It selectively eradicates *C. difficile* while affecting the rest of the microbiota to a lesser extent than vancomycin (Tannock et al., 2010; Krutova et al., 2022). As a result, fidaxomicin is associated with reduced recurrence, but about 1 in 7 patients still relapse following treatment (Louie et al., 2011). Patients with recurrent CDI may benefit from tapered/pulsed antibiotic regimens or monoclonal antibodies (Wilcox et al., 2017; Johnson et al., 2021). However, neither therapies target “dysbiosis,” the root cause of the problem. Since the key to curing the infection lies in the intact gut microbiota, researchers and clinicians are implementing a two-pronged approach, involving antibiotics to remove the pathogen, followed by biotherapeutics to replenish the gut microbiota.

Microbiota protects against *C. difficile* by competing for nutrients, activating immunity, producing antibiotics, or modulating the gut metabolome (Horvat and Rupnik, 2018; Rosa et al., 2018). Additionally, the composition of the gut community influences disease severity. For example, bacteria capable of fiber degradation and bile acid metabolism were linked to less severe diseases. Meanwhile, some bacterial groups, including *Escherichia*, *Streptococcus*, *Enterococcus*, *Helicobacter*, and *Klebsiella*, were associated with worse infection outcomes (Schubert et al., 2015; Lesniak et al., 2022). Much is known about the colonization resistance conferred by the gut microbiota; however, only recent studies have started to reveal the interactions between specific gut bacterial species and *C. difficile*. The microbe-microbe interactions will be the topic of the first section below. In the second section of this review, I will discuss live biotherapeutics for CDI.

## Microbe-microbe interactions

### Bile acid metabolism

*C. difficile* spores must germinate into vegetative cells to colonize the gut and cause disease. Both processes: spore germination and vegetative growth, are influenced by bile acids (Schäffler and Breitrück, 2018). Primary bile acids are those synthesized by the liver and secreted into the intestinal lumen, where they are metabolized into secondary bile acids by the gut microbiota. While primary bile acids, such as cholate and taurocholate, stimulate *C. difficile* spore germination, secondary bile acids, such as lithocholate (LCA) and deoxycholate (DCA), inhibit the vegetative growth of *C. difficile* (Sorg and Sonenshein, 2008, 2010; Heeg et al., 2012; Thanissery et al., 2017).

The transformation from primary to secondary bile acids requires 7α-dehydroxylation. Only a few gut bacterial species have such activity; one of the best-studied is *Clostridium scindens* (Studer et al., 2016; Solbach et al., 2018). By combining metagenomic analyses and mathematical modeling, Buffie and colleagues identified a positive correlation between *C. scindens* and *C. difficile* resistance in clinical samples and mice (Buffie et al., 2015). In addition, adoptively transferring *C. scindens* to *C. difficile*-susceptible mice led to reduced pathogen burden, milder weight loss, and improved survival. *C. scindens*-mediated protection depends on bile acids since pretreatment of *C. scindens*-spiked intestinal content with a bile acid sequestrant abolished its *C. difficile* inhibitory capacity, while engraftment of *C. scindens* in gnotobiotic mice deficient in 7α-dehydroxylation restored DCA and LCA and delayed *C. difficile* expansion (Buffie et al., 2015; Studer et al., 2016). In agreement with these results, the *bai*CD gene cluster, which encodes a key enzyme in bile acid 7α-dehydroxylation, is less prevalent in fecal samples from CDI patients than samples from *C. difficile* negative individuals (Solbach et al., 2018).

Besides its role in secondary bile acid biosynthesis, *C. scindens* secretes 1-acetyl-β-carboline, a tryptophan-derived antibiotic; its antimicrobial activity against *C. difficile* is enhanced in the presence of DCA or LCA (Kang et al., 2019). The dual *C. difficile*-inhibitory mechanism and its effectiveness in animal models make this 7α-dehydroxylating bacterium and related *Clostridium* species promising probiotic candidates for CDI. However, Amrane and colleagues detected *C. scindens* in *C. difficile*-positive stool samples, indicating *C. scindens*, on its own, may not inhibit *C. difficile* in patients and need additional microbial components to protect against CDI (Amrane et al., 2018).

### Microbial-derived nutrients

The gut microbiota also influences *C. difficile* expansion and pathogenesis via microbial-derived nutrients, especially following antibiotic treatments. Gut microbes, such as *Bacteroides thetaiotaomicron*, can cleave sialic acids from the mucosal glycoconjugates but lack the catabolic enzymes to consume the sugar, thereby supplying nutrients for others in the gut lumen (Martens et al., 2008). While the gut microbiota efficiently consumes sialic acids in healthy individuals, antibiotic treatments disrupt this equilibrium, resulting in a transient excess of sialic acids (Ng et al., 2013). *C. difficile*, by upregulating the sialic acid catabolic pathway, can utilize the now available sialic acids for growth and expansion. Similarly, perturbing the gut microbiota chemically or by antibiotics leads to a transient spike in microbiota-derived succinate levels in mice. *C. difficile* adapts to the metabolic shift, induces a pathway to metabolize succinate to butyrate, and gains a competitive advantage (Ferreyra et al., 2014).

In adult and pediatric patients, *Enterococci* positively correlate with *C. difficile* burden and susceptibility (Ozaki et al., 2004; Auchtung et al., 2020; Smith et al., 2022). Consistent with the clinical observations, *Enterococci* are associated with more severe CDI in mouse models. Mice colonized with vancomycin-resistant *Enterococcus* prior to *C. difficile* infection showed worse pathology of colonic tissues and increased toxin levels in the cecal content (Keith et al., 2020). In contrast, *C. difficile* colonization is delayed in mice receiving antibiotics that deplete *Enterococci* (Smith et al., 2022). How do *Enterococci* influence CDI susceptibility? *Enterococcus* species, such as *Enterococcus faecalis*, convert arginine to ornithine using the arginine deiminase pathway. The resulting gut lumen featuring high ornithine and low arginine favors *C. difficile* pathogenesis: *C. difficile* ferments ornithine for energy; meanwhile, arginine limitation may provide an environment cue for *C. difficile* to increase toxin production (Pruss et al., 2022; Smith et al., 2022). Other than *Enterococci*, commensal gut bacteria such as *Clostridium sardiniense* and *Paraclostridium bifermentans* can modulate *C. difficile* infection via the same pathway (Girinathan et al., 2021). *C. sardiniense* supplies ornithine to *C. difficile* and is associated with worsened infection outcomes in mice. In contrast, *P. bifermentans* competes for ornithine, protecting mice against lethal *C. difficile* infection.

Dietary sources also influence intestinal amino acid levels and *C. difficile* susceptibility. For example, a soy protein diet increased the abundance of *Lactobacillus* bacteria, which digest soy proteins into available amino acids for *C. difficile*; therefore, mice on a soy protein diet were more susceptible to CDI than mice given a diet that contains casein as a protein source (Yakabe et al., 2022). Future research exploring nutrient crossfeed in CDI patients and their influence on infection outcomes can help identify avenues for new therapeutic interventions.

### Bacteriocins

Bacteriocins are antimicrobial peptides produced by bacteria that can have narrow- or broad-spectrum activities. Several gut bacterial species produce bacteriocins that can directly inhibit *C. difficile*. For example, *Bacillus thuringiensis* DPC 6431, a bacterial strain derived from human feces, produces bacteriocin thuricin CD, which has a narrow-spectrum of activity that kills *C. difficile* while sparing many intestinal commensals (Rea et al., 2010). The lytic activity of thuricin CD involves permeabilization and depolarization of the target cell membrane, likely due to pore formation (Mathur et al., 2017). Similarly, a human gut symbiont, *Ruminococcus gnavus* E1, synthesizes an antimicrobial sactipeptide, Ruminococcin C1 (RumC1). RumC1 demonstrated bactericidal activities against a panel of Gram-positive bacteria, including *C. difficile*, by possibly inhibiting nucleic acid synthesis (Chiumento et al., 2019).

Bacteriocin-mediated killing also occurs among different strains of *C. difficile.* Diffocins are phage tail-like R-type bacteriocins synthesized by *C. difficile* to kill non-self *C. difficile* strains. They act as molecular syringes to puncture cell membranes after binding to the target cell surface receptors, disrupt the membrane potential, and result in bacterial death (Gebhart et al., 2012; Schwemmlein et al., 2018). This potent inter-strain killing mechanism may explain how colonization with non-toxigenic *C. difficile* protects animals against subsequent *C. difficile* challenges (Borriello and Barclay, 1985). Whether these antibacterial peptides are produced *in vivo* and how they contribute to resistance against *C. difficile* in the host remains to be uncovered.

## Microbiota-derived therapies

**Figure 1:**
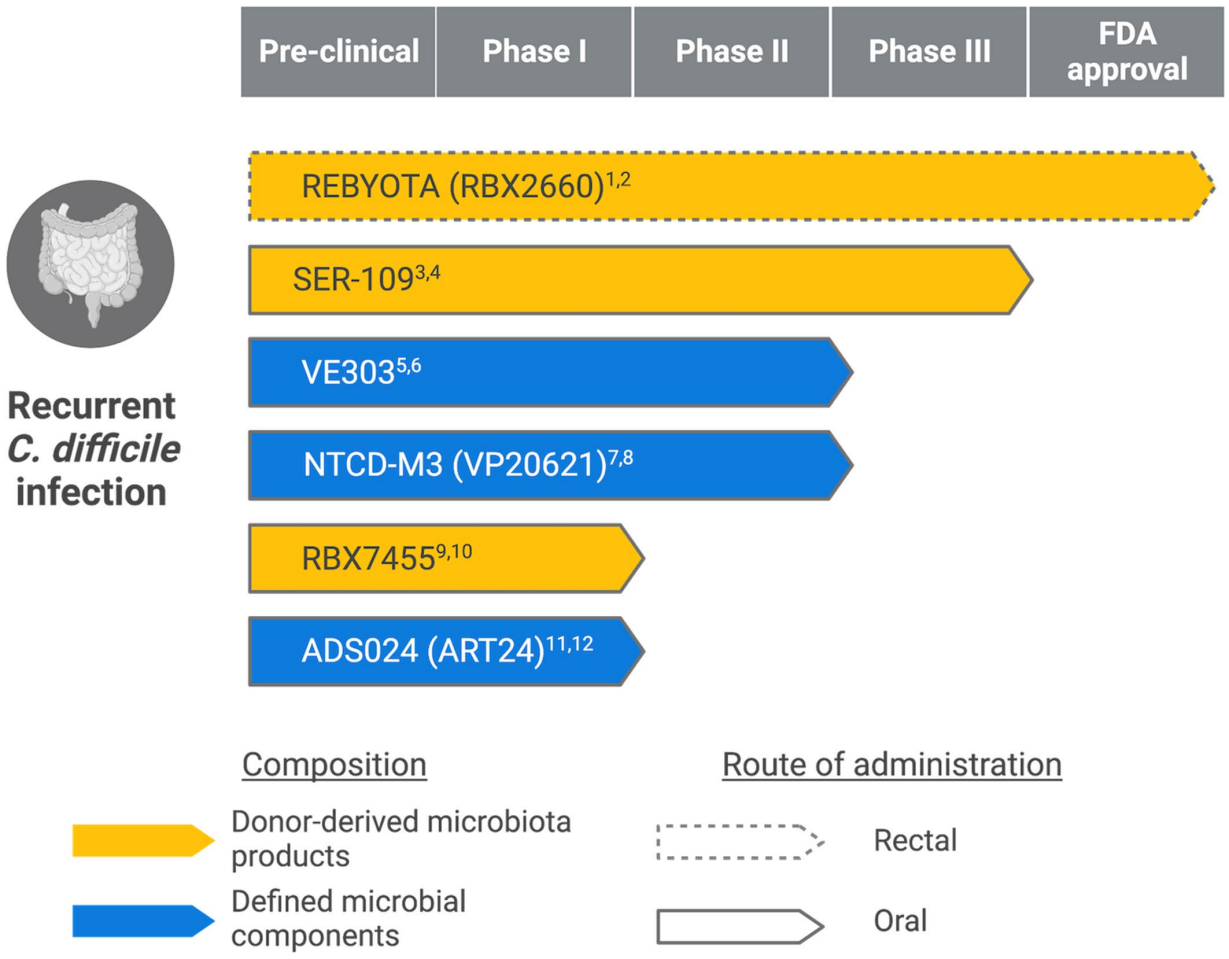
Live biotherapeutic products in different stages of clinical development. (Created with BioRender.com) ^1^ClinicalTrials.gov Identifier: NCT03244644. ^2^https://www.fda.gov/news-events/press-announcements/fda-approves-first-fecal-microbiota-product. ^3^ClinicalTrials.gov Identifier: NCT03183128. ^4^https://www.serestherapeutics.com/our-programs/. ^5^ClinicalTrials.gov Identifier: NCT03788434. ^6^https://www.vedantabio.com/pipeline/ve303. ^7^ClinicalTrials.gov Identifier: NCT01259726. ^8^https://www.destinypharma.com/pipeline/clostridioides-difficile-infections/. ^9^ClinicalTrials.gov Identifier: NCT02981316. ^10^https://www.rebiotix.com/clinical-trials/rbx7455-oral-c-diff-prevention/. ^11^ClinicalTrials.gov Identifier: NCT04891965. ^12^https://adisotx.com/.

Eradicating *C. difficile* requires two actions: killing the vegetative cells and inhibiting spores. While antibiotics, such as vancomycin, are effective at the former, they fail to keep spores at bay: antibiotic-resistant spores can germinate into vegetative cells, produce toxins, and cause colonic inflammation again once antibiotic treatment discontinues (Rupnik et al., 2009). Furthermore, antibiotic treatments also disrupt the normal gut microbiota, alter the gut metabolic state, and leave an opportunity for pathogenic bacteria to thrive (Theriot et al., 2014; Vincent and Manges, 2015; Theriot et al., 2016; Staley et al., 2017; Contijoch et al., 2019). Therefore, probiotic-based therapies that restore the gut microbiota composition and provide sustained protection became attractive alternatives. Figure 1 summarizes live biotherapeutic products (LBPs) for recurrent CDI at various stages of clinical development. They represent two broad categories: donor-derived microbiota products and defined microbial components.

Fecal microbiota transplantation (FMT) involves the transfer of donor microbiota to replenish a recipient’s gut microbial composition. Despite being generally well-tolerated and effective against recurrent CDI, the possible transmission of infectious agents poses safety concerns, especially in the immunocompromised population (DeFilipp et al., 2019; Tariq et al., 2019). Therefore, standardized screening and manufacturing processes were urgently needed. Recently, FDA approved REBYOTA (also known as RBX2660), the first fecal microbiota product for recurrent CDI. REBYOTA is manufactured under a standardized process that includes rigorous pathogen testing to minimize the health risks of FMT. While effective – 70.6% of patients treated with REBYOTA remain free of CDI recurrence within 8 weeks (Khanna et al., 2022); it requires storage at −80°C and is delivered to patients via enema. The same company is developing an oral capsule-based therapy called RBX7455 that contains lyophilized bacteria stable at room temperature (Khanna et al., 2021). If it succeeds in clinical trials and gains regulatory approval, RBX7455 may offer a more convenient option for recurrent CDI treatment.

SER-109 is another donor-derived therapeutic. The manufacturing of SER109 enriches *Firmicute* spores while inactivating potential bacterial, viral, and fungal pathogens (McGovern et al., 2021; Feuerstadt et al., 2022; McChalicher et al., 2022). In a phase III clinical trial (ClinicalTrials.gov Identifier: NCT03183128), subjects dosed with SER-109 were less likely to have recurrent infections following standard-of-care antibiotic treatment than patients in the placebo group (Feuerstadt et al., 2022). These spore-forming *Firmicutes* may mediate protection against *C. difficile* by competing for essential nutrients and modifying bile acid profiles in the gut (Ridlon et al., 2014; Theriot et al., 2016).

Donor-derived microbiota products contain a mixture of microbes and vary in composition (Khoruts et al., 2021); it is, therefore, challenging to correlate clinical efficacy with biological components. In contrast, defined microbial components have standardized compositions and can be rationally designed based on biological functions.

VE303 is a defined bacterial consortium consisting of eight commensal strains of *Clostridium* (Dsouza et al., 2022). In a phase 1a/b study, healthy volunteers dosed with VE303 after vancomycin pretreatment showed accelerated recovery of diverse microbial communities and increased levels of secondary bile acids and short-chain fatty acids associated with colonization resistance against *C. difficile*. VE303 is also safe and well-tolerated in the study subjects. Subsequent clinical trials will evaluate the safety and efficacy of VE303 in preventing recurrent CDI (ClinicalTrials.gov Identifier: NCT03788434).

Researchers have also focused on non-toxigenic *C. difficile* (NTCD), which lacks genes for toxin production and frequently colonizes hospitalized patients (Shim et al., 1998). The initial colonization with NTCD could prevent subsequent toxigenic *C. difficile* infections in animal models and patients (Wilson and Sheagren, 1983; Borriello and Barclay, 1985; Seal et al., 1987). One NTCD strain, NTCD-M3 (previously known as VP20621), has demonstrated safety and efficacy in a phase II clinical trial (Gerding et al., 2015): oral administration of NTCD-M3 spores was safe and well-tolerated and reduced CDI recurrence in patients clinically cured with antibiotics (Villano et al., 2012; Gerding et al., 2015). However, the concern with NTCD is the possibility of gaining toxin-producing genes via horizontal gene transfer (Brouwer et al., 2013); whether this occurs *in vivo* remains unclear and requires close monitoring.

Besides restoring the host microbiota, LBP could act directly on *C. difficile.* ADS024 (formerly ART24) is a single strain LBP of *Bacillus velezensis* isolated from a fecal sample of a healthy donor. ADS024 exhibits dual actions on clinically relevant *C. difficile* strains, including direct inhibition and toxin degradation (O’Donnell et al., 2022; Xie et al., 2023). The product has recently completed a phase I study, which evaluates the safety of ADS024 in recently cured CDI patients (ClinicalTrials.gov Identifier: NCT04891965).

## Discussion

Given the complex interactions between *C. difficile* and the gut microbes, combined treatments targeting the pathogen and the microbiota may yield better clinical outcomes than antibiotic treatments alone. Live biotherapeutics can protect against recurrent CDI by expediting the microbiota recovery, restoring the metabolic profile, mediating colonization resistance, or directly inhibiting *C. difficile* (Gerding et al., 2015; Khanna et al., 2021, 2022; Dsouza et al., 2022; Feuerstadt et al., 2022; O’Donnell et al., 2022). After colonization, the live ingredients in the LBP remain viable and potentially provide sustained protection against enteric pathogens. However, LBP’s non-traditional features also require distinct approaches and considerations in regulatory approval, manufacturing, and prescription (Dreher-Lesnick et al., 2017). Further research to understand the interactions among the introduced microbes, the microbiota, and host immunity will be crucial as these results will inform treatments for enteric infections and other conditions involving the gut microbiota.

## Author contributions

RW conceptualized the manuscript topic, conducted literature research, created the figure, and wrote the manuscript.

## Funding

This publication was supported by the Princeton University Library Open Access Fund.

## Conflict of interest

The author declares that the research was conducted in the absence of any commercial or financial relationships that could be construed as a potential conflict of interest.

## Publisher’s note

All claims expressed in this article are solely those of the authors and do not necessarily represent those of their affiliated organizations, or those of the publisher, the editors and the reviewers. Any product that may be evaluated in this article, or claim that may be made by its manufacturer, is not guaranteed or endorsed by the publisher.
